# Exposure to respirable silica contributes to lower airway inflammation in asthmatic horses

**DOI:** 10.1111/jvim.17160

**Published:** 2024-10-17

**Authors:** Alessandra Romolo, Giulia Costa, Beatrice Sica, Giulia Memoli, Matteo Ardit, Francesco Di Benedetto, Donata Bellis, Silvana Capella, Elena Belluso, Michela Bullone

**Affiliations:** ^1^ Department of Veterinary Sciences University of Turin, Largo Paolo Braccini 2‐5 Turin 10095 Italy; ^2^ Department of Earth Sciences University of Turin, Via Valperga Caluso 35 Turin 10125 Italy; ^3^ Department of Physics and Earth Sciences University of Ferrara, Via Saragat 1 Ferrara 44122 Italy; ^4^ Interdepartmental Centre for Studies on Asbestos and Other Toxic Particulates “Giovanni Scansetti”, University of Turin, Via Pietro Giuria, 7 Turin 10125 Italy

**Keywords:** bronchoalveolar lavage, dusts, lung inflammation, respirable crystalline silica, respirable fraction

## Abstract

**Background:**

Respirable mineral particles can induce lower airway inflammation, but the role they play in asthma of horses is unknown.

**Objectives:**

Respirable mineral particles, particularly respirable silica, are an overlooked determinant of chronic lung inflammation (asthma) in horses.

**Animals:**

Twenty‐three horses from an equine hospital population: 11 moderately affected (MEA), 7 severely asthmatic (SEA), and 5 control horses free from respiratory clinical signs.

**Methods:**

Prospective observational study. The quantity and quality of mineral particles found in bronchoalveolar lavage fluid (BALF) were characterized, with particular attention to silica content. Polarized light microscopy performed on cytospin slides identified intracellular birefringent particles as silica. Spectrometry‐based analysis performed on whole BALF determined total mineral and silica percentage and concentration. Group‐related differences in BALF mineral and silica load were investigated as well as associations with BALF cytology.

**Results:**

Intracellular birefringent particles were increased in SEA vs MEA (median [interquartile range, IQR]), 12 [7] vs 4 [5] particles/30 high power fields [hpf], respectively; *P* = .01) and vs controls (4 [2] particles/30 hpf; *P* = .02). Total mineral concentration in BALF was similar between the groups studied, whereas silica concentration and percentage were increased in SEA vs MEA (1758 [887] particles/mL and 20 [10]% vs 867 [662] particles/mL and 8 [6]%; *P* = .009 and *P* = .001) and control group (355 [330] particles/mL and 6 [3]%; *P* = .0003 and *P* = .002). Silica load in BALF was associated with BALF neutrophilia in MEA and SEA.

**Conclusions and Clinical Importance:**

Respirable silica is associated with neutrophilic lower airway inflammation in horses and might contribute to asthma development.

**Abbreviations**
ACVIM: American College of Veterinary Internal Medicine
ANOVA: analysis of variance
BAL: bronchoalveolar lavage
BALF: bronchoalveolar lavage fluid
BSD: backscattered electron detector
EDTA: ethylenediaminetetraacetic acid
GMC: giant multinucleated cells
GMC:M: giant multinucleated cell:macrophage
hpf: high power field
MEA: mild to moderate equine asthma
pp: particles
RCS: respirable crystalline silica
SEA: severe equine asthma
SEM‐EDS: scanning electron microscope‐energy dispersive x‐ray spectroscopy

## 1 INTRODUCTION

The pathophysiology of asthma in horses (EA) is related to air quality as well as exposure to dust and pollutants that can reach the most distal airways and alveoli, inducing inflammation.^1^ Previous work has elucidated the role of endotoxins, molds, and fungal spores or elements as inhaled determinants of lower airway inflammation in horses.^2^, ^3^, ^4^, ^5^ However, this list is likely incomplete, and the pathobiology of EA is still considered unclear.

Air is naturally contaminated by mineral dusts originating from natural sources (ie, rock erosion by wind and other meteorological factors) or anthropogenic activities (eg, industrial processes), some of which can cause lung damage.^6^ The toxic potential of mineral dusts on the respiratory system is mainly determined by their size, shape, and mineral composition, although other physicochemical factors likely are involved.^7^ Mineral dusts with diameter <5 μm, referred to as respirable mineral particles, are considered most dangerous because they can reach the alveoli and cause inflammatory or toxic responses.^8^ Concerning shape, elongated particles with a bidimensional axis ratio >3 : 1 are considered more harmful.^9^, ^10^ As for mineral composition, although the study of pulmonary mineral toxicology is still in its infancy with many compounds having unknown or incompletely proven toxic potential,^6^ some compounds have known toxicity, such as respirable crystalline silica (RCS).^11^, ^12^

Silica (SiO_2_) is very abundant on earth, present mainly in rocks and sand.^13^ Case series of progressive fibrotic lung disease described as silica‐induced pneumoconiosis, as well as other silica‐induced diseases, have been reported in horses of all ages living in regions where the soil is naturally rich in silica, such as California.^12^, ^14^, ^15^ These studies indicate that horses, as well as human beings, are sensitive to silica‐induced lung damage. Surfaces used for horse riding and racing around the world are composed of silica in varying percentages, which can reach nearly 100% in certain instances.^16^ Previous work has shown that respirable dust in riding centers is rich in RCS,^17^ and some reports in the literature suggest that exposure to the equestrian riding environment, especially in the absence of dust control measures, may affect human health and lead to chronic lung disease.^18^, ^19^ We hypothesized that respirable mineral particles, particularly RCS, are an overlooked determinant of chronic lung inflammation in horses. Specifically, our hypothesis was that mineral particles and RCS would be increased in the bronchoalveolar lavage fluid (BALF) of asthmatic as compared to non‐asthmatic horses, and that they would correlate with BALF inflammation. In detail, we aimed to: (a) characterize the quantity and quality of respirable mineral particles found in the BALF of asthmatic and non‐asthmatic horses selected from a cohort of horses referred to a veterinary teaching hospital, and (b) assess the relationship between BALF mineral particle load and cytology. Besides RCS, the toxicity of which to the respiratory tract is well‐known, we also focus on feldspars and plagioclases, based on available information on the toxic potential of these mineral species.^6^

## 2 MATERIALS AND METHODS

### 2.1 Study design

A prospective study was conducted from April 2021 to November 2022. Asthmatic and non‐asthmatic horses were selected from clinical cases referred to the Veterinary Teaching Hospital of the University of Turin. Horses with a final diagnosis of EA, either mild to moderate or severe, as defined by the revised American College of Veterinary Internal Medicine (ACVIM) consensus statement,^1^ and non‐asthmatic control horses were recruited as described below. All the horses studied underwent a respiratory examination and bronchoalveolar lavage. The BALF samples underwent were submitted for conventional cytology using brightfield and polarized light microscopy to assess for inflammation and the presence of birefringent mineral particles, as well as scanning electron microscope‐energy dispersive x‐ray spectroscopy (SEM‐EDS) for qualitative and quantitative assessment of respirable mineral load. The study protocol was approved by the Ethics and Animal welfare Committee of our Institution (Prot. N. 711, March 17, 2021). Written informed consent was obtained from the owners.

### 2.2 Horse group definition

Asthma cases were recruited among horses referred to our Veterinary Teaching Hospital for respiratory problems or examinations if they were finally diagnosed with severe or mild to moderate EA (SEA and MEA groups, respectively), defined according to the revised ACVIM consensus statement.^1^ Briefly, MEA cases consisted of horses with chronic respiratory signs (eg, cough, nasal discharge, increased breathing effort during or after exercise, poor performance) and increased BALF cell counts in the absence of increased respiratory effort at rest. The cutoffs used to define BALF increased cell counts were >5% for neutrophils, >5% for mast cells, and >1% for eosinophils. These cutoffs were established based on average cytological counts of our referral equine population free of respiratory signs. The SEA horses were defined as horses with a history of recurrent episodes of increased respiratory effort at rest reversible by corticosteroid treatment or environmental changes aimed at decreasing antigen exposure, as reported by the owners and confirmed by the referring veterinarian. Horses with SEA were included regardless of their disease status and BALF cytology results at the time of clinical assessment (exacerbation or remission).

Control cases were recruited from the equine hospital population and defined based on the absence of relevant respiratory signs as assessed by the attending clinician during clinical examination and confirmed by the owner and referring veterinarian, along with the absence of increased cell counts on BALF cytology. For control cases, respiratory examination and BALF cytology were proposed as additional clinical tests without charge, if clinically unnecessary, and performed only with the owner consent. During clinical examination, respiratory effort was measured using a previously validated weighted clinical score in all recruited horses.^20^

Fever, altered respiratory pattern caused by respiratory conditions other than EA (eg, pneumonia, pleuropneumonia, strangles) or non‐respiratory conditions (eg, cardiac disease, severe anemia), current or previous (last 2 weeks) administration of corticosteroids, and having undergone surgery in the previous 7 days were considered exclusion criteria.

### 2.3 Bronchoalveolar lavage procedure and cytology

Bronchoalveolar lavage procedures were performed on standing sedated horses, as previously described.^21^ Briefly, two 250‐mL boluses of 0.9% warm sterile saline solution were instilled into the distal airways using a catheter (BAL300 Large Animal Bronchoalveolar Lavage Catheter, MILA International, Florence, USA) and gently withdrawn. A 4‐mL aliquot of the resuspended sample was submitted for cytology in an EDTA tube to minimize cell clumping, and the remaining portion of BALF was retained in 50‐mL plastic tubes and stored at room temperature for subsequent SEM‐EDS analysis. Two 200‐μL aliquots of unfiltered BALF were centrifugated at 500 rpm for 10 minutes within 2 hours from sampling and stained with May‐Grunwald‐Giemsa for cytospin slide preparation (MirastainerTM II System, EMD Chemicals Inc., Germany). Cell counts were performed by the same operator, blindly, on ≥5 randomly selected high power fields (hpf, 400×) and ≥500 inflammatory cells, expressed as a percentage. Epithelial cells were excluded from the count. Cell counts were validated by an experienced pathologist.

### 2.4 Mineral particle assessment in BALF by polarized light microscopy

Cytospin slides were cleaned to remove any residual dust and assessed by polarized light microscopy by the same operator who performed the BALF cell counts, blinded to the horse identification. Determining the nature of a respirable‐sized mineral particle by polarized light microscopy is challenging because no universally accepted criteria are available, especially in the absence of pathognomonic histological lesions. Based on extensive experience with pneumoconiosis assessment by a human medical pathologist involved in our study (DB), and in accordance with the available literature on the subject, 2 criteria were used to define silica‐like particles on polarized light microscopy and to distinguish them from non‐silica‐like particles based on size and birefringence color. Silica‐like particles were defined as particles ≤5 μm in diameter, faintly to brightly birefringent, with a milky to slightly bluish birefringence color and halo. Of note, amorphous silica, characterized by lower pathogenicity compared with RCS, is characterized by a lack of birefringence because of its unstructured form,^22^ and cannot be identified by polarized microscopy. Other particles (mainly mineral but also organic in nature) often were larger, with a blue, reddish, yellow, pink or bright white birefringence. Particles with mixed birefringence patterns also were classified as other particles. Particles were classified as intracellular (with details of the inflammatory cell in which they were identified) or extracellular, based on their localization within or outside of the cell cytoplasm. Mineral particle counts were performed over 30 randomly selected hpf (400× magnification) per slide, representing approximately one‐third (30%‐35%) of the cytospin area, and expressed as particles/30 hpf. To account for different cell densities, intracellular particle counts also were calculated as particles/BALF total nucleated or macrophage cell count.

### 2.5 Mineral particle assessment in BALF by SEM‐EDS

A 20 mL aliquot of BALF was chemically digested with 5 mL sodium hypochlorite (NaClO) at approximately 40°C for at least 48 hours to eliminate organic matrix. The suspension was filtered using a cellulose ester filter (25 mm diameter, 0.45 μm porosity) to retain inorganic particles. Dried filters were mounted on stubs with conductive bio‐tape and coated with a thin layer of carbon (metallization) to add conductivity. Inorganic particle analysis was performed blindly, at 4000× magnification, using a Jeol JSM‐IT300LV SEM‐EDS instrument equipped with a microanalysis Inca Energy 200 cones INCA X‐act SDD EDS detector. Samples were investigated after a regular layout for a total time of 4 hours regardless of the total area studied, using a backscattered electron detector (BSD). Particle diameters (major or longitudinal and minor or cross‐sectional) were obtained from SEM bidimensional images. Mean diameter was calculated as the mean value of the 2 diameters measured. Diameter ratio was calculated as the ratio of the major and minor diameters. A diameter ratio >3 defines a fiber rather than a particle,^9^ and is associated with increased toxicity.^10^ Semi‐quantitative information on a particle's chemical composition was obtained using EDS microanalysis. The SEM‐EDS spectra obtained were compared with those in the internal database of the laboratory of the Department of Earth Sciences for mineral species identification. A chemical analysis of the membrane also was performed for each sample to exclude any potential chemical interference. The particle concentration of each sample was expressed per 1 mL of BALF (pp/mL) and calculated using the formula:

$$\text{BALF mineral particle concentration}\;\left(\mathrm{pp}/\mathrm{mL}\right)=\frac{\frac{{\mathrm{pp}}_{\mathrm{oss}}\left(n\;\mathrm{PP}\right)\;\times \;A_{\mathrm{tot}}\;\left({\mathrm{\mu m}}^{2}\right)}{A_{\mathrm{oss}}\;\left({\mathrm{\mu m}}^{2}\right)}}{\mathrm{Vol}\;\left(\mathrm{mL}\right)},$$

where, pp_oss_ is the number of observed particles, *A*_tot_ is the total area of the filter assessed, *A*_oss_ is the area of the filter which has been observed, and Vol is the total volume of BALF analyzed.

### 2.6 Statistical analysis

Statistical analysis was performed using STATA Statistical Software: Release 15 (StataCorp LLC, College Station, Texas, USA). A priori power analysis was not conducted because of a lack of relevant data in the available literature. Data distribution was tested using the D'Agostino and Pearson omnibus normality test. Except for raw particle data (presented as mean ± SD), continuous variable data are summarized as median [25th;75th percentile]. Raw particle data (mean particle diameter and diameter ratio) were averaged for each horse and this independent variable was used for subsequent statistical analysis. Differences among the 3 groups studied were assessed using the Kruskal‐Wallis test with Dunn's post‐test. Bonferroni correction was applied for repeated measures. A posteriori, sensitivity analyses were conducted for silica particle‐associated data to assess robustness of the results.^23^ Sensitivity analyses included assessing the impact of outliers, assessing the impact of removing SEA horses in disease remission, and running a sensitivity power analysis with G* Power software.^24^ Relationships between lower airway inflammation‐related variables (BALF neutrophil, mast cell, eosinophil, macrophage, and lymphocyte counts; BALF giant multinucleated cell:macrophage (GMC:M) ratio; BALF absolute cell counts; dependent variables) and BALF mineral particle‐associated variables (intracellular silica particles, intracellular other particles, extracellular silica particles, other extracellular particles, BALF silica concentration and percentage, BALF feldspars concentration and percentage, BALF plagioclase concentration and percentage, BALF total mineral particle concentration, independent variables) were assessed using univariate linear regression models, with the exception of those variables for which age confounding was deemed relevant based on previous knowledge and statistical testing, for which bivariate linear regression was employed. Adjusting for age was considered appropriate because both the studied exposure (mineral‐particle associated variables, and in particular pulmonary silica load) and the studied outcome (inflammation‐associated variables, and in particular SEA and associated neutrophilia) previously have been shown to be significantly associated with age (confounding). To this aim, the relationship between age (independent variable) and both lower airway inflammation‐related and BALF mineral particle‐associated variables (dependent variables) was investigated using univariate linear regression. The Benjamini‐Hochberg correction was used to adjust for multiple comparisons. Within each group (controls, MEA, SEA), the correlation between BALF neutrophilia and silica‐related variables (intracellular silica particles, BALF silica concentration and percentage) was studied using a 2‐tailed Pearson or Spearman correlation test, based on data distribution. Alpha was set at 0.05 for all analyses. The data that support the findings of our study are available from the corresponding author (MB), upon reasonable request.

## 3 RESULTS

### 3.1 Horses

The clinical details of the 23 horses studied are summarized in Table 1. Briefly, 7 horses were diagnosed with SEA, 11 with MEA, and 5 were classified as non‐asthmatic controls. Horses with SEA were significantly older than those with MEA, but not control horses. Sex distribution was similar in all groups studied. Cytologically, MEA horses had neutrophilic (n = 4), mastocytic (n = 2), eosinophilic (n = 1), mixed neutrophilic‐mastocytic (n = 2), mixed neutrophilic‐eosinophilic (n = 1), or mixed neutrophilic‐mastocytic‐eosinophilic (n = 1) inflammation (Table 2). Five horses with SEA in exacerbation had BALF neutrophilia at the time of clinical examination, which was associated with eosinophilia (2%) in 1 case. Both SEA horses in clinical remission had normal BALF cytology, were kept outdoors, and displayed mild clinical signs.

**TABLE 1:** Details of the horses studied.

	All	Controls	MEA	SEA
Horse (n)	23	5	11	7
Age^#^ (years)	12 [5.5, 16]	7 [3, 25]	8 [4.5, 11]*	17 [14.5, 20.5]
Sex (M : F)	18 : 5	2 : 3	10 : 1	6 : 1
Weighted clinical score (range: 2‐8)	2 [2, 3]	2 [2, 2]	2 [2, 2]	6 [3, 6]
Complaint: cough (n)	7	0	4	3
Complaint: poor performance (n)	5	0	1	4
Complaint: dyspnea during/after exercise (n)	13	0	6	7
Complaint: altered breathing pattern at rest (n)	7	0	0	7
SEA exacerbation at the moment of examination (n)	5	‐	‐	5

*Note*: Data are reported as median [25th, 75th percentile].

Abbreviations: MEA, mild to moderate equine asthma; RR, respiratory rate; SEA, severe equine asthma.

^#^ Significant difference among the groups (Kruskal‐Wallis test; *P* < .05).

* Significantly different from SEA (Dunn's post‐test; *P* = .01).

**TABLE 2:** Lung inflammation data.

	All	Controls	MEA	SEA
Horses (n)	23	5	11	7
Instilled BAL volume (mL)	500	500	500	500
Recovered BAL volume^#^ (mL)	250 [140, 400]	‐	450 [400, 500]*	160 [120, 215]
Total BALF nucleated cell count (μL)	293 [220, 486]	125 [102, 148]	340 [293, 486]	240 [206, 687]
BALF neutrophils^#^ (%)	6.5 [2, 20]	1 [0.5, 2]*	6.5 [2, 18]*	22 [4, 52]
BALF macrophages^#^ (%)	50 [28, 57]	54 [52, 57]*	50 [26, 61.5]	30 [25, 46]
BALF mast cells^#^ (%)	3 [1, 5.2]	4 [3, 5]*	4 [1, 7]*	1 [0.5, 3]
BALF lymphocytes (%)	36 [26.5, 47]	36 [36, 42.5]	35.5 [26, 47]	34 [26.5, 65.5]
BALF eosinophils (%)	0 [0, 1]	0 [0, 0]	0 [0, 2]	0 [0, 1]
BALF GMC (GMC:M ×10^3^)	7.9 [4.1, 16]	4.1 [0, 5.4]	7.9 [2.6, 16]	14 [7.7, 24]

*Note*: Data are reported as median [25th, 75th percentile].

Abbreviations: BALF, bronchoalveolar lavage fluid; GMC, giant multinucleated cells; GMC:M, giant multinucleated cell:macrophage ratio; MEA, mild to moderate equine asthma; SEA, severe equine asthma.

^#^ Significant difference among the groups (Kruskal‐Wallis test; *P* < .05).

* Significantly different from SEA (Dunn's post‐test; *P* < .05).

### 3.2 Mineral particle load in equine BALF

**FIGURE 1:**
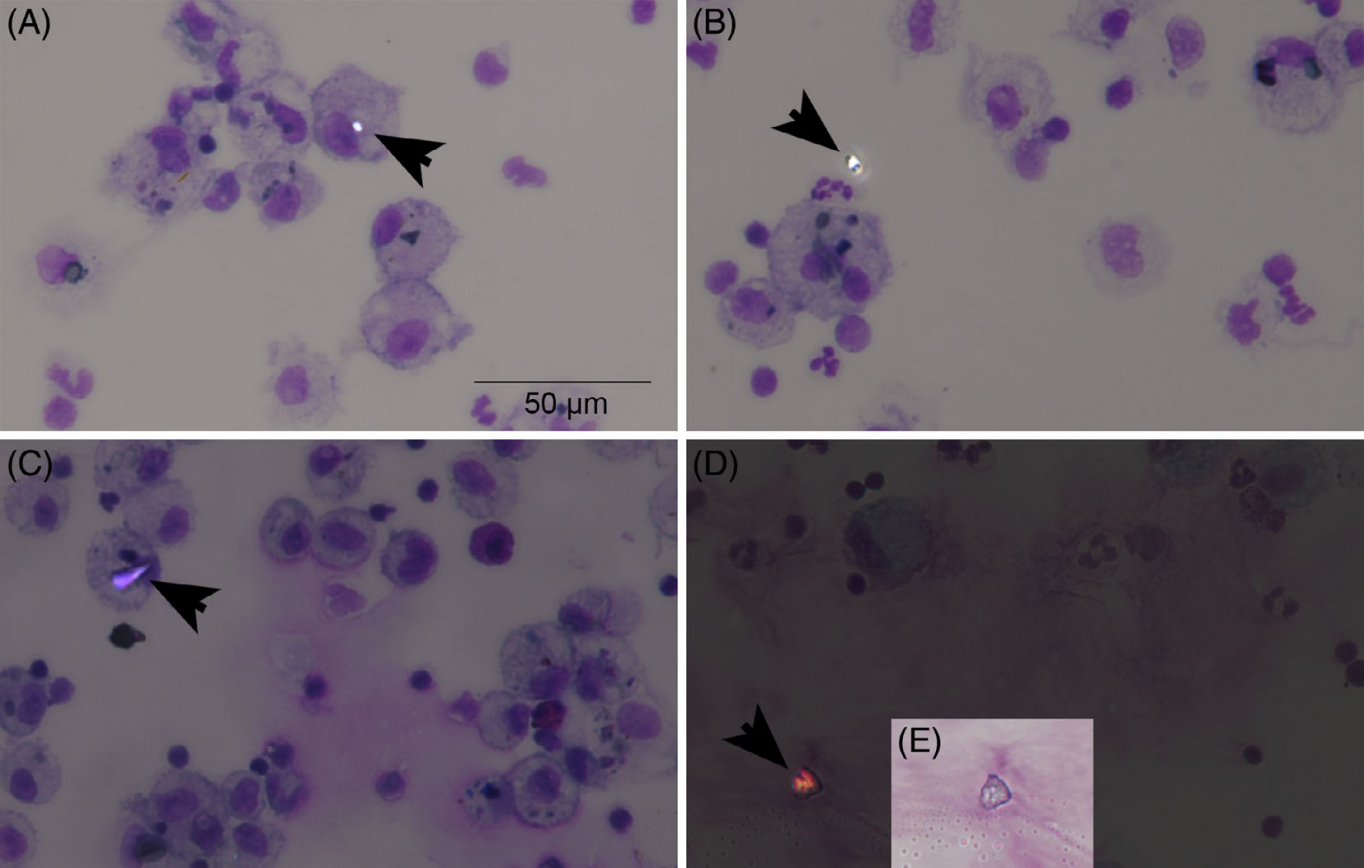
Representative examples of birefringent mineral particles in equine bronchoalveolar lavage fluid (BALF). (A) Intracellular silica‐like particle. (B) Extracellular silica‐like particle. (C) Other intracellular particle. (D) Other extracellular particle. Images were obtained from BALF cytospin slides stained with May Grunwald‐Giemsa and visualized with polarized light microscopy at 400× magnification. Panel (E) shows the birefringent particle in (D) using brightfield microscopy at the same magnification.

**FIGURE 2:**
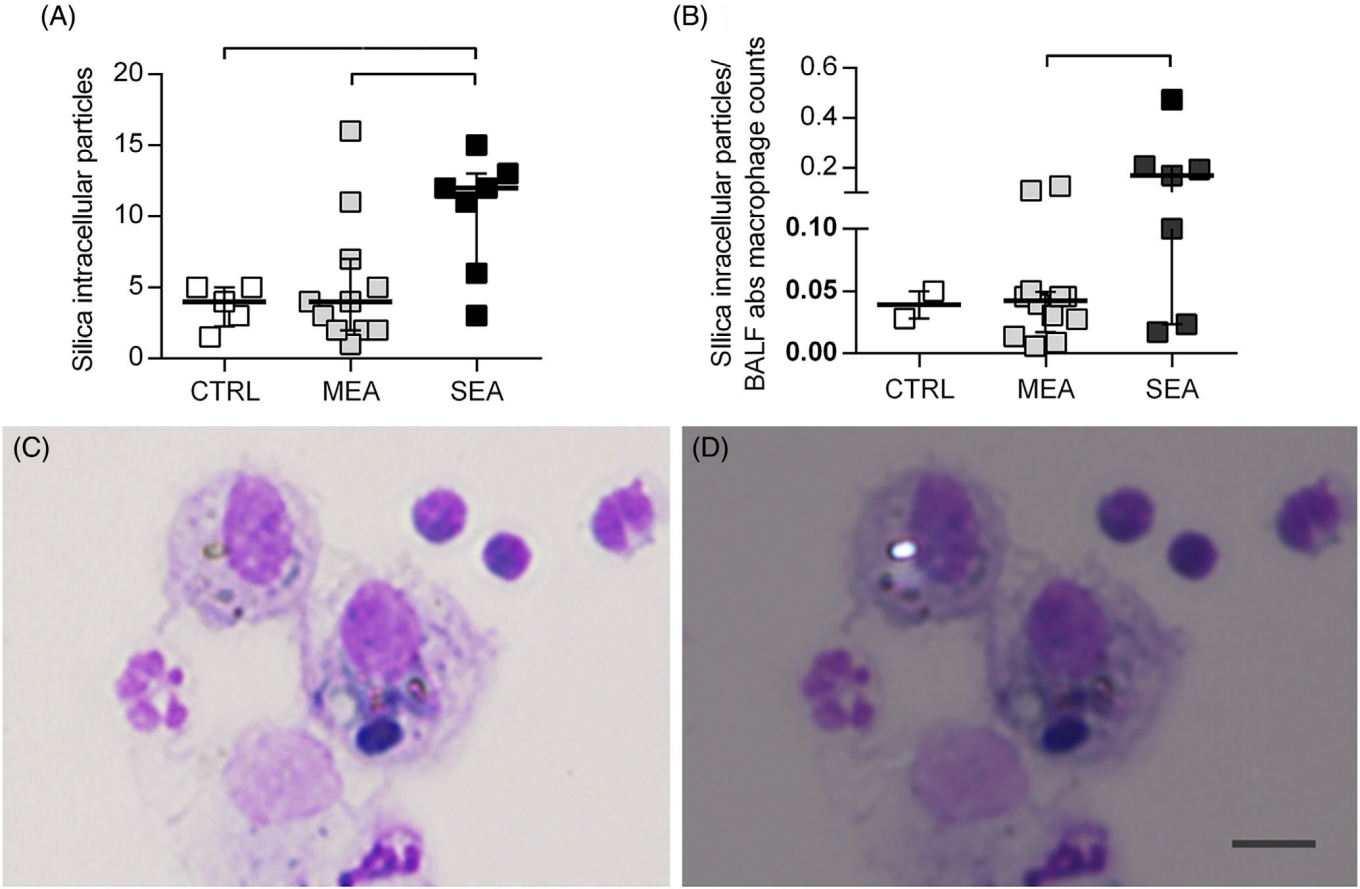
Silica‐like intracellular particle load assessed by polarized light microscopy on May Grunwald‐Giemsa‐stained cytospin slides. Panels (A) and (B) show the results of silica‐like intracellular particles/30 hpf and silica‐like intracellular particles/bronchoalveolar lavage fluid absolute macrophage counts, respectively. Lines represents median values and bars interquartile range. Panels (C) and (D) show a representative example at brightfield (C) and polarized microscopy (D) of a silica‐like intracellular particle. Images were taken at 400× magnification. Solid line indicates significant differences between groups (*P* < .05). Scale bar: 10 μm.

**FIGURE 3:**
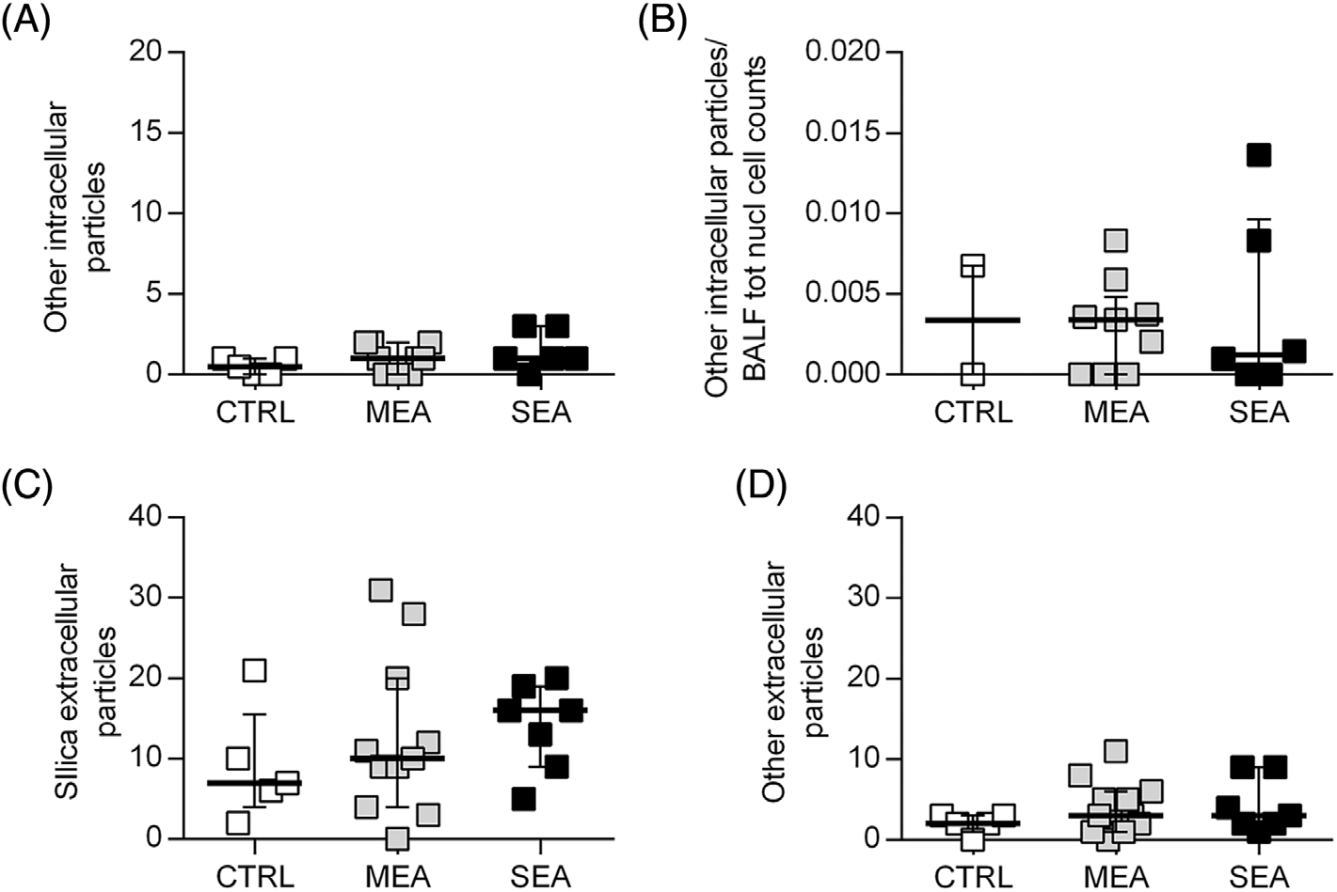
Mineral particle load assessed by polarized light microscopy on May Grunwald‐Giemsa‐stained cytospin slides. (A) Other intracellular particles/30 hpf. (B) Other intracellular particles/bronchoalveolar lavage fluid total nucleated cell counts. (C) Silica‐like extracellular particles. (D) Other extracellular particles. Lines represent median values and bars interquartile range.

Particle counts performed using polarized light microscopy on BALF cytospin slides identified birefringent particles compatible with our definition of silica‐like as well as other particles, both within and outside cell cytoplasm (Figure 1). Most of the observed intracellular particles (148/169, 88%) matched our definition of silica‐like particles. They were observed mainly within macrophages (117/148, 79%), giant multinucleated cells (GMC; 30/148, 20.3%), and only rarely within neutrophils (1/148, 0.7%). The number of intracellular silica‐like particles differed between the examined groups (Figure 2). Post hoc tests indicated that intracellular silica‐like particles/30 hpf were increased in SEA compared with MEA (median [25th, 75th percentile] 12 [6, 13] vs 4 [2, 7] particles/30 hpf, respectively; *P* = .01) and to control horses (4 [3, 5] particles/30 hpf; *P* = .02; Figure 2A). When intracellular silica‐like particles were corrected by BALF absolute total nucleated cell counts, the difference observed between SEA and MEA was confirmed (0.041 [0.010, 0.058] vs 0.008 [0.005, 0.025] particles/BALF total nucleated cell count [Mann Whitney test, *P* < .05]). The same was true when correcting for the absolute number of macrophages (Figure 2B). No difference was observed between the groups in the number of extracellular silica‐like particles (SEA: 16 [9, 19], MEA: 10 [4, 20], controls: 7 [6, 10] particles/30 hpf; *P* = .5) as well as other intracellular (SEA: 1 [0.75, 3], MEA: 1 [0, 2], controls: 1 [0, 1] particles/30 hpf; *P* = .5) and extracellular particles (SEA: 3 [2, 9], MEA: 3 [1, 6], controls: 2 [2, 3] particles/30 hpf; *P* = .6; Figure 3).

**FIGURE 4:**
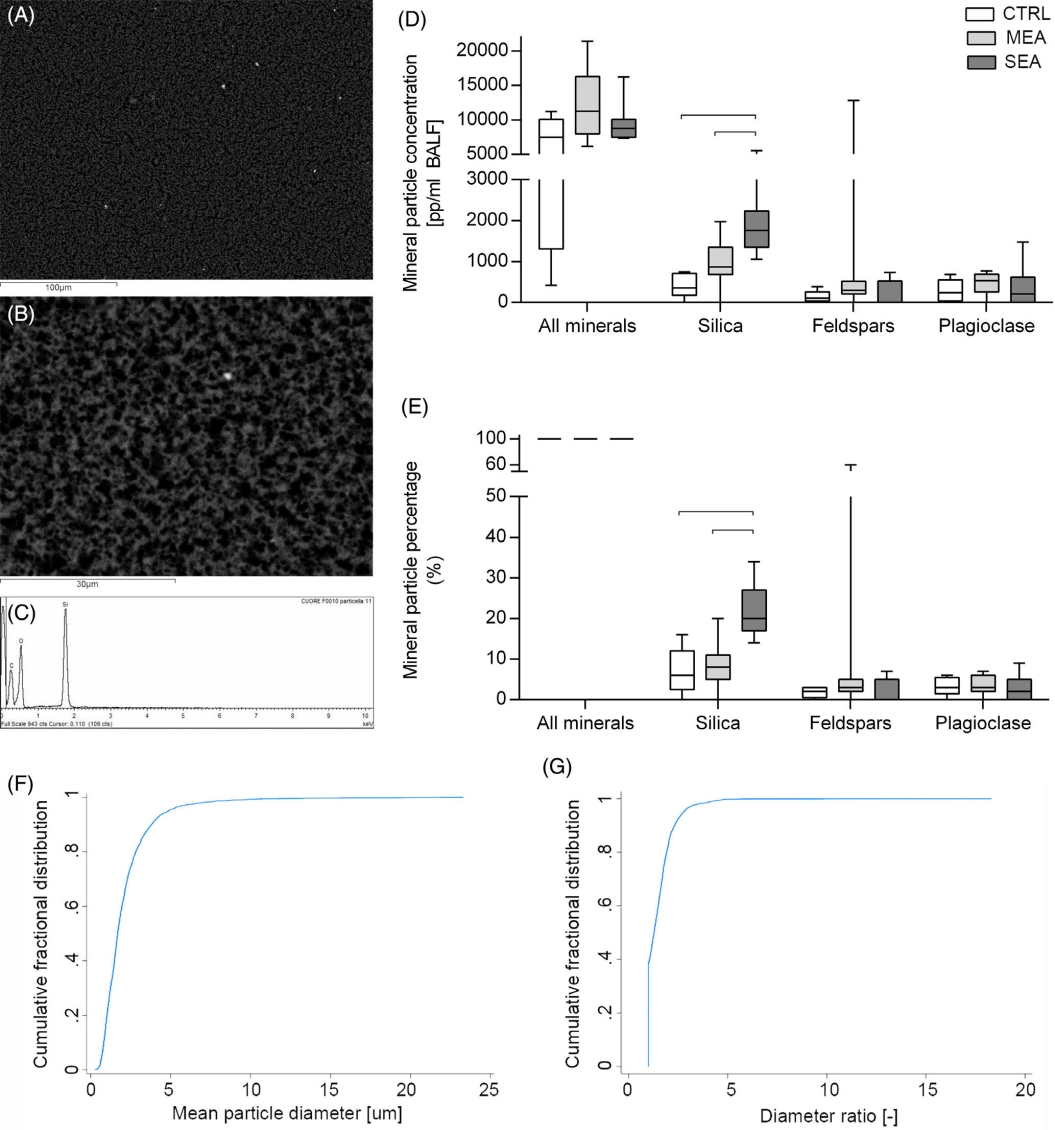
Mineral particle load assessed by SEM‐EDS analyses. Panels (A) and (B) show SEM images obtained at 500× and 2000× magnification, respectively. Scale bars correspond to 100 μm (A) and 30 μm (B). Panel (C) shows the chemical spectrum of the mineral imaged in 2B, composed by silica and oxygen, and identified as a silica particle. Panels (D) and (E) show mineral particle concentrations and percentage in bronchoalveolar lavage fluid (BALF), respectively. Boxes depict 25‐50‐75 percentiles and whiskers represent minimum to maximum values. Solid line indicates significant differences between groups (*P* < .05). Panels (F) and (G) show the cumulative distribution frequency plots of mineral particle mean diameter (F) and diameter ratio (G) of all BALF studied, as assessed by SEM‐EDS analyses.

Total mineral particle counts performed using SEM‐EDS on BALF samples identified total particle concentrations ranging from 429 to 21 404 pp/mL BALF, with no significant differences between groups (SEA: 8759 [7470, 10 092], MEA: 11 267 [7972, 16 269], controls: 7454 [1311, 10 070] pp/mL BALF; *P* = .1; Figure 4). A total of 2314 particles were analyzed singularly. The mean diameter ± SD of mineral particles was 2.15 ± 1.77 (range, 0.3‐23.3) μm, with 95% particles having a mean diameter <5 μm (Figure 4F). Mineral particle mean diameter ratio was 1.52 ± 0.83 (range, 1‐18), with 96.5% particles having a diameter ratio ≤3 (cutoff for defining a fiber; Figure 4G). Both mean diameter of mineral particles and their diameter ratio were similar among the groups studied (Table 3).

**TABLE 3:** Raw mineral particle data obtained by SEM‐EDS on BALF samples.

Group	All minerals			Silica (SiO_2_)			Feldspars			Plagioclase		
	n obs	D mean^#^ [μm]	D ratio [−]	n obs	D mean [μm]	D ratio [−]	n obs	D mean^#^ [μm]	D ratio [−]	n obs	D mean [μm]	D ratio [−]
All (23)	2314	2.15 ± 1.77	1.52 ± 0.83	284	2.19 ± 2.10	1.50 ± 0.63	166	2.69 ± 3.12	1.48 ± 0.58	93	2.27 ± 1.16	1.62 ± 0.58
SEA (7)	693	2.28 ± 1.84	1.53 ± 0.67	153	2.24 ± 2.23	1.48 ± 0.64	18	1.49 ± 0.55	1.24 ± 0.38	29	2.30 ± 1.06	1.59 ± 0.44
MEA (11)	1374	2.09 ± 1.74	1.51 ± 0.88	109	2.14 ± 2.08	1.49 ± 0.65	137	2.88 ± 3.38	1.52 ± 0.60	50	2.41 ± 1.29	1.63 ± 0.64
CTRL (5)	247	1.97 ± 0.97	1.57 ± 0.69	22	2.05 ± 0.90	1.66 ± 0.49	11	2.17 ± 1.23	1.40 ± 0.60	14	1.68 ± 0.63	1.61 ± 0.70

*Note*: D mean and D ratio are expressed as mean ± SD.

Abbreviations: CTRL, control horses; D mean, mean particle diameter; D ratio, mean particle diameter ratio; MEA, mild to moderate equine asthma; n obs, number of observed particles; SEA, severe equine asthma.

Silica (SiO_2_), as determined by pattern recognition of the chemical spectrum, was the 2nd most abundant mineral compound identified in the BALF samples studied, after a group of minerals called tectosilicates. A total of 284 particles over 2380 assessed (12%) were identified as silica, 162 (7%) as feldspars and 96 (4%) as plagioclase (Table 3). Differences were observed for silica particle concentration and percentage among the groups studied (Figure 4A‐E). Silica particle concentration and percentage were higher in SEA compared to control (1758 [1350, 2237] particles/mL and 20 [17, 27]% vs 355 [177, 713] particles/mL BALF and 6 [2.5, 12]%; *P* = .001 and *P* = .007, respectively) and MEA (867 [684, 1112] particles/mL and 8 [5, 11]%; *P* = .03 and *P* = .005). No differences in particle mean diameter and diameter ratio were observed when silica, feldspars, and plagioclase particles were studied individually (Table 3).

### 3.3 Relationship between respirable mineral particle load and inflammation in the equine lung

After adjusting for age, neutrophil counts in BALF were significantly and positively associated with BALF silica concentration and percentage as determined by SEM‐EDS (Table 4).

**TABLE 4:** Association between BALF neutrophilia, mineral load‐related variables, and age.

	BALF neutrophils [%] (dependent variable)	Age (independent variable)
Intracellular silica‐like particles	***1.83 (0.60;3.06); P = .02***	0.21 (−0.02;0.44); *P* = .07
Intracellular other particles	6.10 (−1.13;13.34); *P* = .32	0.04 (−0.01;0.09); *P* = .14
Extracellular silica‐like particles	0.45 (−0.39;1.31); *P* = 1.00	0.22 (−0.21;0.65); *P* = .31
Extracellular other particles	1.47 (−0.76;3.71); *P* = .9	0.04 (−0.12;0.21); *P* = .58
Total mineral particle concentration (pp/mL BALF)	0.00 (−0.00;0.00); *P* = 1.00	−65.0 (−333.3;201.3); *P* = .61
Silica particle concentration (pp/mL BALF)	***0.01 (0.00;0.01); P = .002***	**60.5 (5.1;115.9); *P* = .03**
Silica particle BALF percentage	***1.17 (0.37;1.97); P = .01***	**0.60 (0.22;0.98); *P* = .003**
Feldspars particle concentration (pp/mL BALF)	0.00 (−0.00;0.00); *P* = 1.00	3.3 (−141.6;148.3); *P* = 1.0
Feldspars particle BALF percentage	0.14 (−0.44;0.72); *P* = 1.00	−0.00 (−0.67;0.66); *P* = 1.0
Plagioclase particle concentration (pp/mL BALF)	0.01 (−0.11;0.28); *P* = 1.00	−1.8 (−21.1;17.6); *P* = .8
Plagioclase particle BALF percentage	−0.25 (−3.03;2.54); *P* = 1.00	−0.02 (−0.16;0.12); *P* = .8
BALF neutrophils (%)	‐	**0.80 (0.06;1.53); *P* = .03**

*Note*: Results are expressed as *β* coefficient (95% CI). *P*‐values of the associations between BALF neutrophilia and mineral‐load related variables are adjusted for multiple comparisons (Benjamini‐Hochberg correction). Italics indicate analyses adjusted for age using bivariate linear regression. Bold indicates significant associations. On average, and keeping age constant when adjusting for this variable, for each 1‐unit increase in the independent variable (either in row or column), the dependent variable is expected to change by the *β* coefficient.

Abbreviations: BALF, bronchoalveolar lavage fluid; pp, particle(s).

Macrophage counts in BALF were significantly and positively associated only with BALF plagioclase percentage as assessed by SEM‐EDS (*β* coefficient: 5.03; 95% confidence interval [CI]: 2.15, 7.91; *P* = .02). No significant associations were observed between other variables of lower airway inflammation (BALF mast cell, eosinophil, lymphocyte cell counts, GMC:M ratio, and absolute cell count) and any mineral species‐associated variable (data not shown).

In SEA, BALF neutrophilia significantly correlated with both BALF silica concentration and BALF silica percentage (*r*_*s*_ = 0.82, *P* = .03 and *r*_*s*_ = 0.81, *P* = .03, respectively). In MEA, BALF neutrophilia correlated with silica intracellular particle counts/BALF total nucleated cell count (*r*_*p*_ = 0.84, *P* = .005), whereas its relationship with silica intracellular particle counts/30 hpf was not significant (*r*_*s*_ = 0.58, *P* = .06).

### 3.4 Post hoc sensitivity analyses

Sensitivity analysis identified a horse with MEA with 16 BALF silica intracellular particles count/30 hpf as the only outlier in the dataset studied. Removal of the outlier decreased the variance (MEA without outlier: 3.5 [2, 5.5] particles/30 hpf), and the difference between MEA and SEA groups remained (*P* = .01). Overall, data interpretation remained unchanged. The outlier was retained in the dataset because there was no reason to suspect the unexpected value was caused by a technical error. Removing the 2 horses with SEA in disease remission from the dataset also did not alter the overall results or their interpretation (differences between the groups and correlations were maintained). Post hoc sensitivity power analyses were run for all tests performed on silica‐associated variables and assuming that the observed effects (all defined as large, *f* > 0.40) correspond with true effects. Results indicated that data derived from BALF silica percentage could be regarded as the most robust in our dataset, because our study design had sufficient power (≥0.8) to detect differences as those observed or even differences 10% smaller between the groups.

## 4 DISCUSSION

By means of polarized light microscopy, we showed that birefringent silica‐like intracellular particles are increased in the BALF of SEA compared to MEA and control horses, even after correcting for BALF total nucleated cell counts and despite a similar number of total particles on the slide. Spectroscopy analyses provided specific chemical identification of BALF mineral particles and further confirmed this result, emphasizing increased silica particle concentration and percentage in SEA compared to MEA and controls, always despite a similar concentration of total mineral particles in the 3 groups. Lastly, BALF silica load appeared positively associated with BALF neutrophil numbers in our population even when adjusting for age and when assessed in MEA and SEA separately. In the complex setting of EA pathophysiology, our study provides the 1st evidence to support a possible role of inhaled mineral particles, especially silica, as an additional determinant of lower airway inflammation in horses.

Silica is highly abundant in the environment and is a major component of most footing surfaces designed for horses, with particles reaching respirable size (<5 μm in diameter). The pulmonary toxicity of silica is related to its physical and chemical properties which, in addition to high biopersistence,^13^, ^25^, ^26^, ^27^ leads to dysregulation of the immune system. Alveolar macrophages exposed to RCS particles are activated but unable to properly process this mineral species. Silica‐activated macrophages produce reactive oxygen species and chemokines which, via inflammasome activation, lead to recruitment and activation of neutrophils and mast cells.^28^, ^29^, ^30^ These cells appear not to be phagocytic for minerals based on our observations, because all silica‐like particles were observed within macrophages. Inhaled RCS generate a chronic and self‐perpetuating proinflammatory environment within the lungs, with clinical effects most often seen 5 to 20 years after initial exposure in humans who develop silicosis.^13^, ^31^, ^32^ The significant association observed in our study between age and many silica‐associated variables suggests that silica biopersistence is relevant in horses and that this mineral accumulates within equine lungs over time. Even if silica does not primarily cause development of EA, it might contribute to worsening of the disease over time or to episodes of disease exacerbation by both local and systemic immune‐mediated effects.^33^ In support of this hypothesis, data in the human medical literature suggest that silica exposure might cause both asthma and silicosis.^34^ Experimental models support an adjuvant role for silica exposure in the exacerbation of asthma‐related inflammation.^35^, ^36^, ^37^, ^38^, ^39^ In vitro work has shown that silica can induce acute responses in macrophages, within a few hours after exposure, although silica‐induced disease presentation classically is defined as a chronic disease. Two studies in horses also used silica microspheres together with inhaled fungal spores and lipopolysaccharide to induce EA exacerbation and to study the in vitro response of alveolar macrophages.^40^, ^41^ The individual contribution of each component of the challenge material used was not determined, however, and a possible synergistic effect should not be excluded. A further clue of the potential role of inhaled silica in SEA might be the fact that high temperatures have been shown to induce disease exacerbations,^42^ which might be linked with increased airborne respirable mineral particles or dusts during dry periods.

Silica load in equine BALF was significantly associated with BALF neutrophilia in the population studied, both when all groups were studied together, and when MEA and SEA groups were studied individually. Although a clustering effect might have confounded the joint analysis, within‐group analyses showed significant correlations between BALF neutrophilia and silica intracellular particle counts in MEA, and between BALF neutrophilia and BALF silica concentration and percentage in SEA. These findings might be explained by differences in the sensitivity of the methods used to detect silica particles, coupled with differences in disease chronicity between MEA and SEA, and silica clearance dynamics. Available data from murine models suggest that silica retention half‐life in the lungs is 2 to 24 months, depending on exposure dose and time, with initial accumulation in alveolar macrophages and subsequent increases in extracellular free silica associated with parenchymal accumulation and macrophage dysfunction.^26^ In MEA, when disease chronicity spans weeks to months,^1^ the observed relationship between BALF neutrophilia and intracellular silica particles may depict the initial phase of the silica‐induced immune pro‐inflammatory response. In SEA, when disease chronicity spans years,^1^ altered phagocytosis, recruitment, or macrophage dynamics in BALF occurring over time^21^, ^26^, ^43^ may hinder the relationship between BALF neutrophilia and intracellular silica particles, whereas the relationships between BALF neutrophilia and silica concentration and percentage in BALF become apparent. Alternatively, amorphous silica accumulation in SEA may explain the difference observed. Silica occurs in 2 forms in nature, crystalline and amorphous, and only the former is visible as a birefringent particle by polarized light microscopy,^22^ whereas both are detected by SEM‐EDS because they have the same chemical composition and spectra. Lastly, the small sample size of our study might have accounted for this discrepancy.

The silica load of BALF was higher in SEA compared with MEA and control groups, regardless of the method or units used to quantify it. Because BALF is a sample inherently characterized by an unknown dilution factor, the possibility that our data were biased by the larger BALF volumes obtained in control and MEA (more diluted samples) vs SEA horses was considered. However, if such were the case, we would have expected also to detect differences in other non‐silica particles studied as well as total mineral particles, but this effect was not observed. The fact that silica was increased in SEA vs MEA and controls also when assessed in relative terms (percentage), further excludes the possibility that results were biased by the BALF dilution factor. Lastly, the volume of BALF fluid recovered was not associated with BALF total or silica mineral particle load or with BALF total nucleated cell counts (data not shown), which argues against the presence of a bias related to BALF dilution in the data reported. Because of silica has high biopersistence in the organism, where it accumulates over time,^26^ attention has been paid to applying a robust statistical approach that could consider and adjust for the possible confounding effect of aging. Adjusted results confirmed the significant positive association between BALF silica load (exposure) and SEA (outcome or disease). Taken together, although our data do not prove the causality of silica in the pathogenesis of SEA, they do show that silica accumulates in the lower airways (and possibly pulmonary parenchyma) of horses with SEA to a larger extent than in age‐matched horses with MEA or those without clinically relevant respiratory disease.

Mineral particle size and shape are 2 important determinants of the toxic and pro‐inflammatory potential of silica and must be fully characterized in respiratory toxicology studies. The BALF mineral particle size and shape were overall similar in the groups studied. Two things need to be acknowledged in this regard. First, the proposed approach for mineral particle size and shape description is approximate, because it is bidimensional and dependent on the random position the particle assumes while depositing on the filter. Second, particle toxicity often is related to its surface rather than its volume, which cannot be determined properly with this type of measurement. Consequently, the majority of the particles observed were within the micron scale and of respirable size, in agreement with the sampling site.

Mechanisms potentially leading to higher accumulation of silica particles in the lungs of SEA horses remain speculative and could occur before or after disease presentation. Conditions leading increased environmental exposure, decreased pulmonary clearance of respirable mineral particles, or both could contribute equally. In this regard, evidence in the literature supports high levels of respirable silica exposure in riding arenas^17^, ^19^ as well as decreased ability of severely asthmatic lungs to clear large inhaled particles during periods of disease exacerbation.^44^ Although environmental exposure during exercise activities may appear questionable because of its short duration, peaks of marked silica exposure could be as relevant or even more relevant than the same cumulative distribution of silica over a longer period of time.^45^ Furthermore, epithelial dysregulation, bronchospasm, mucus accumulation, small airway closure, overall respiratory system heterogeneity and possibly other mechanisms involved in SEA^1^ could contribute to the enhanced silica accumulation observed in this group. Silica itself impairs macrophage phagocytic ability,^28^ thus potentially participating in a feedback loop.

Control horses included in our study were defined as such based on their clinical presentation and cytology, in the absence of systematic rebreathing bag examination. As for cytology, the higher cutoff reported in the literature (5%) was used for mast cell percentage. This decision was based on cytological findings from the referral equine population of our hospital, in which mast cell counts >2% are commonly observed, even in the absence of associated respiratory disease, and the reasons of which remain unexplained. Given the role of mast cells in innate immunity and as 1st line responders, mild to moderate increases in BALF mast cell percentage, especially in the absence of high total nucleated cell counts (<300 cells/μL), could be interpreted as a transient physiological response of the lung to environmental stimuli.^46^, ^47^ We cannot exclude however the possibility of subclinical disease in the control horses, because 4 of 5 of them did not exercise at high intensity.

Epidemiological evidence suggests that other mineral particles may be involved in the complex pathogenesis of EA because of their cytotoxic, pro‐inflammatory, or even adjuvant properties. Feldspars and plagioclase (calcium‐rich feldspar), for example, have been associated with adverse biological health effects, although evidence is limited to in vitro experimental data.^6^, ^48^, ^49^ Our findings however do not support a relevant role for these mineral species in lower airway inflammation of horses. Their pathologic effects, if any, may be ascribed to their role as foreign bodies in the lung.

Our study had some limitations. First, its observational nature does not allow for conclusions about causality concerning the role of silica in lung inflammation and disease. However, given the potential toxicity linked to prolonged silica exposure, experimental evidence should 1st be sought in alternative non‐animal models. Second, cytology data on mineral and silica‐like particles are inherently imprecise. Some minerals, and silica itself in its amorphous form, do not show birefringence under polarized light and could have been missed. Also, minerals with birefringence similar to that of silica might have been misinterpreted. Third, the number of horses studied per group was low, especially controls. This limitation is particularly relevant given the observational nature of the study, in which many uncontrolled (and possibly still unknown) factors could have influenced the results. Lastly, the clinical score used has low sensitivity in horse populations with mild clinical signs, for which other more specific scores^50^ should be employed in future work.

In conclusion, respirable silica is associated with neutrophilic lower airway inflammation in asthmatic horses and might contribute to the development and progression of EA. Direct cytotoxic and pro‐inflammatory effects of silica as well as its dysregulating effects on the systemic immune response are well‐described in the medical literature, and their role in EA deserves further attention. Given the reported prevalence of silica in riding and racing surfaces designed for horses, further work on this topic is warranted to explore airborne exposure to mineral particles during horse exercise to safeguard the respiratory health of horses.

## CONFLICT OF INTEREST DECLARATION

Authors declare they have no conflict of interest.

## OFF‐LABEL ANTIMICROBIAL DECLARATION

Authors declare no off‐label use of antimicrobials.

## INSTITUTIONAL ANIMAL CARE AND USE COMMITTEE (IACUC) OR OTHER APPROVAL DECLARATION

Approved by the Ethics and Animal Welfare Committee of the Department of Veterinary Sciences of the University of Turin (Prot. N. 711, March 17, 2021).

## HUMAN ETHICS APPROVAL DECLARATION

Authors declare human ethics approval was not needed for this study.
